# *Cladophora glomerata* methanolic extract promotes chondrogenic gene expression and cartilage phenotype differentiation in equine adipose-derived mesenchymal stromal stem cells affected by metabolic syndrome

**DOI:** 10.1186/s13287-019-1499-z

**Published:** 2019-12-17

**Authors:** Lynda Bourebaba, Izabela Michalak, Meriem Baouche, Katarzyna Kucharczyk, Krzysztof Marycz

**Affiliations:** 1 Department of Experimental Biology, Faculty of Biology and Animal Science, Wrocław University of Environmental and Life Sciences, Norwida 27B, 50-375 Wrocław, Poland; 2 International Institute of Translational Medicine, Jesionowa, 11, Wisznia Mała, 55-114 Malin, Poland; 3 Department of Advanced Material Technologies, Faculty of Chemistry, Wrocław University of Science and Technology, Smoluchowskiego 25, 50-372 Wrocław, Poland; 4 Collegium Medicum, Institute of Medical Science, Cardinal Stefan Wyszyński University (UKSW), Wóycickiego 1/3, 01-938 Warsaw, Poland

**Keywords:** *Cladophora glomerata*, EMS, ASCs, Chondrogenesis, Hypertrophy, microRNA

## Abstract

**Background:**

Chondrogenesis represents a highly dynamic cellular process that leads to the establishment of various types of cartilage. However, when stress-related injuries occur, a rapid and efficient regeneration of the tissues is necessary to maintain cartilage integrity. Mesenchymal stem cells (MSCs) are known to exhibit high capacity for self-renewal and pluripotency effects, and thus play a pivotal role in the repair and regeneration of damaged cartilage. On the other hand, the influence of certain pathological conditions such as metabolic disorders on MSCs can seriously impair their regenerative properties and thus reduce their therapeutic potential.

**Objectives:**

In this investigation, we attempted to improve and potentiate the in vitro chondrogenic ability of adipose-derived mesenchymal stromal stem cells (ASCs) isolated from horses suffering from metabolic syndrome.

**Methods:**

Cultured cells in chondrogenic-inductive medium supplemented with *Cladophora glomerata* methanolic extract were experimented for expression of the main genes and microRNAs involved in the differentiation process using RT-PCR, for their morphological changes through confocal and scanning electron microscopy and for their physiological homeostasis.

**Results:**

The different added concentrations of *C. glomerata* extract to the basic chondrogenic inductive culture medium promoted the proliferation of equine metabolic syndrome ASCs (ASCs_EMS_) and resulted in chondrogenic phenotype differentiation and higher mRNA expression of collagen type II, aggrecan, cartilage oligomeric matrix protein, and *Sox9* among others. The results reveal an obvious inhibitory effect of hypertrophy and a strong repression of *miR-145-5p*, *miR-146-3p*, and *miR-34a* and *miR-449*a largely involved in cartilage degradation. Treated cells additionally exhibited significant reduced apoptosis and oxidative stress, as well as promoted viability and mitochondrial potentiation.

**Conclusion:**

Chondrogenesis in EqASCs_EMS_ was found to be prominent after chondrogenic induction in conditions containing *C. glomerata* extract, suggesting that the macroalgae could be considered for the enhancement of ASC cultures and their reparative properties.

## Introduction

Chondral and osteochondral lesions resulting from traumatic injury or other more complex pathologies are among the most prevalent conditions in horses and humans and usually result in the development of osteoarthritis and progressive deterioration of joints [1]. Articular cartilage is a highly elastic and extremely resistant tissue to various constraints, repeated loads, and consequent solicitations. Lacking from any blood, nervous, and lymphatic network, cartilage is a tissue with significantly reduced regenerative capacity in case of injury or damage. In the horse, the occurrence of cartilaginous lesions represents a serious issue regarding the athletic performances for the equine industry. Moreover, the occurrence of developmental defects such as osteochondrosis can lead to the appearance of osteochondritis dissecans [2].

Matrix networks, otherwise extracellular matrix (ECM), consist essentially of chondrocyte cells that maintain cartilage homeostasis by continuously synthesizing and degrading matrix components, in response to environmental stimuli such as growth factors, cytokines, and biomechanical changes. During postnatal remodeling of the cartilaginous epiphysis, chondrocytes develop as a result of the aggregation and differentiation of mesenchymal precursors, associated with cascades of molecular events such as expression of *Sox-5*, *Sox-6* and *Sox*-*9*, and the secretion of the main matrix components, namely collagens II, VI, IX, and XI, binding proteins, hyaluronically linked proteoglycans (HA), aggrecan, and versican [3]. Formation of the cartilaginous ring continues with the secondary organization of chondrocytes in both proliferative and quiescent zones. Cells that are in continuous proliferation respond to prehypertrophy and hypertrophy differentiation programs to become hypertrophic cells characterized by overexpression of key hypertrophic markers such as *Runx2*, collagen X, and alkaline phosphatase. These molecular modifications are accompanied by calcification of the matrix and emergence of cells that positively express certain factors such as *VEGF* and osteocalcin, resulting in vascular invasion, chondrocyte apoptosis, and trabecular bone deposition [4]. During pathological conditions, collagen is often degraded following the action of certain enzymes belonging to the family of collagenases, while aggrecan can be degraded by matrix metalloproteinases (MMPs) or by aggrecanases [5, 6].

Although cartilage damage is often attributed to traumatic injury, a number of different pathologies have also been linked to the pathophysiological mechanism leading to the degradation of cartilage tissue. More recently, the involvement of certain metabolic disorders such as obesity and metabolic syndrome has been demonstrated [7]. Meta-inflammation, often observed during the development of metabolic syndrome, is thus triggering many dysfunctions affecting the synthesis and action of various key metabolic factors such as adipokines, cytokines, supplements, lipids, and vitamin D [8]. Metabolic overload can initiate the oxidative stress, and thus contribute to the onset of chronic inflammation triggering to a cascade of molecular reactions that leads to cellular dysfunction [9]. The presence of abnormally high levels of pro-inflammatory cytokines including interleukin (IL)-1β, IL-6, IL-8, and tumor necrosis factor alpha (TNF-α), baked at the recruitment and activation of the nuclear factor -κB (NF-κB) signaling pathway, that modulates subsequently the catabolic activity of articular chondrocytes and initiate the extracellular matrix degradation process via upregulation of MMPs expression [10].

It is now widely accepted that MSCs play a pivotal role in the repair and regeneration of damaged cartilage; this has largely been attributed to their high capacity for self-renewal, their pluripotency, and their multiple anti-inflammatory and immunomodulatory effects [11]. Although cartilage is largely composed of chondrocytes, these later originate from the differentiation of chondroblasts that develop from MSCs; newly formed chondrocytes subsequently secrete extracellular matrix components and become trapped in it [12]. It has been demonstrated that during their chondrogenic differentiation, MSCs are prone to highly express genes of key components involved in cartilage replacement, namely type II collagen, aggrecan, and *Sox9* [13]. Moreover, the paracrine properties of MSCs also seem to play a critical role; thus, these cells can modulate the expression of several growth factors mostly derived from the *TGF-β* superfamily, anti-inflammatory mediators, and anticatabolic molecules that may potentiate the stem cell-mediated regeneration of the cartilage. In addition, it has been evidenced that mesenchymal stem cells derived from adipose tissue exert a repressor effect on MMP-13 expression, thus potentially inhibiting collagen degeneration in pathological cartilage [14]. Although MSCs represent an innovative and effective therapeutic strategy for the management of various degenerative diseases, it has been shown that therapeutic potential of cell therapy can be seriously affected by certain existing pathological conditions. Thus, aging and metabolic disorders are the main conditions that could cause severe disturbances at the genomic, epigenomic, and proteomic levels, impairing the various functionalities of MSCs. It has been shown that the proliferative, differentiating, and paracrine signaling abilities of those cells may be deteriorated in case of diabetes, metabolic syndrome, or cardiovascular disorders, thus limiting the regenerative potential of MSCs [15, 16]. Equine metabolic syndrome (EMS), which belongs among the most common endocrine diseases, refers to a constellation of clinical abnormalities that are mainly associated to insulin resistance (IR). Moreover, EMS has been strongly linked to obesity, chronic inflammation of the adipose tissue, and high risk of laminitis development [17, 18]. Many studies have shown for example that adipose-derived stromal stem cells (ASCs) derived from equine metabolic syndrome horses are quite dysfunctional. Indeed, ASCs_EMS_ are prone to high apoptotic tendency concomitantly to a reduced proliferative potential and marked downregulation of stemness genes such as *Oct-4*, *Nanog*, and *Sox2.* Moreover, these cells exhibit a significant increase in ROS production resulting from dominant oxidative stress, the onset of endoplasmic reticulum stress, and collapse of mitochondrial activity [17–23]. As a result, the evidence of the use of adjuvant molecules that would allow the improvement and potentiation of therapeutic effects of stem cells seems to be increasingly confirmed. It is now widely accepted that natural compounds possess strong stem cell stimulatory properties mainly proliferation and differentiation of MSCs [24]. *Cladophora glomerata* is freshwater green macroalga, which grows naturally in eutrophic water ecosystems, forming dense, seasonal mats. As other algae, and apart from having a high nutritive value, it also contains various bioactive compounds including pigments (carotenoids, chlorophylls, sulphated polysaccharides, amino acids, vitamins, and polyphenols). These compounds are known to protect the algal cells against stressful conditions, such as ultraviolet radiation, temperature changes, and fluctuation in nutrient [25]. On the other hand, algal phenolic compounds were reported to exhibit anti-gastric ulcer, anti-inflammatory, analgesic, hypotensive, antibacterial, anticancer, and antioxidant activities in vitro and in vivo [26]. Moreover, some studies have also pointed out the positive effects of *C. glomerata* extracts on EMS ASCs through viability, proliferation, and mitochondrial potential improvement and the reduction of apoptosis, oxidative, and ER stress. The same extract has also alleviated deleterious effect of H_2_O_2_ on equine ASCs while stimulating the own antioxidant defenses of challenged cells [19, 27]. Taking into account the fact that the macroalga *Cladophora glomerata* has already largely proved its effectiveness in improving the molecular condition of ASCs derived from EMS horses, as well as a wide range of other biological benefits with, in particular, anti-inflammatory abilities, the investigation of an eventual potentiation of the chondrogenic differentiation properties of ASCs isolated from horses suffering from metabolic syndrome was undertaken in this study.

## Materials and methods

All chemicals and cell culture reagents were obtained from Sigma Aldrich (Taufkirchen, Germany), unless otherwise stated.

### Algal biomass

The biomass of freshwater macroalgae—*Cladophora glomerata—*was collected by hand from the surface of the pond in Tomaszówek, Łódź Province, Poland (51° 27′ 21″ N 20° 07′ 43″ E) in October 2016. No specific permissions were required for this location/activity. The harvesting of algae was carried out on a private land. This study did not involve endangered or protected species. Then the biomass was air-dried and fine milled using a grinding mill (Retsch GM 300, Germany) [17].

### Extraction of the algal biomass

The biomass (5 g) was extracted with 250 mL of methanol (Avantor Performance Materials Poland S.A. Gliwice, Poland) by shaking in a shaker at 200 rpm (IKA KS 260 basic) for 48 h in the darkness [28]. After filtration through a filter paper, the solvent was evaporated on an evaporator (BUCHI Labortechnik AG, Rotavapor R-100, Switzerland). The remaining dry residue was then dissolved in 40 mL of methanol (Avantor Performance Materials Poland S.A. Gliwice, Poland). The experiment was performed in two replications and resulted in an extraction yield of 5.06 ± 0.06%. Phytochemical composition analysis of the *Cladophora glomerata* dried biomass was previously carried out and results described by Marycz et al. [19].

### Equine ASC isolation and cell culture

Adipose tissue samples were collected from the tail base area of adult healthy and EMS horses, under local anesthesia induced by 2% lidocaine (Polfa S.A., Warsaw, Poland). Samples were amply washed using Hanks’ Balanced Salt Solution (HBSS) supplemented with 1% antibiotics to avoid microbial contamination. Tissues were then finely minced using a surgical blade, digested by means of collagenase type I solution (0.1 mg/mL) during 40 min at 37 °C and 5% CO_2_, and centrifuged afterwards at 1200×*g* for 10 min. The obtained cell pellet was resuspended in Dulbecco’s modified Eagle’s medium (DMEM) containing 1000 mg/L glucose supplemented with 5% of fetal bovine serum (FBS), and 1% of a penicillin and streptomycin (PS) solution in culture flasks. Cultures were maintained in a humidified CO_2_ incubator (37 °C, 5% CO_2_, 95% air atmosphere), passaged every 3 days (80–90% of confluence) using a trypsin-EDTA solution (TrypLE Express, Life Technologies) and cells were used at third passage for experiments.

Cellular purity was confirmed using fluorescent-activated cell sorting technique (BD FACSCalibur, Becton Dickinson, Franklin Lakes, New Jersey, USA). ASC phenotyping was assessed by flow cytometry analysis using fluorochrome conjugated monoclonal antibodies (anti-CD105, Acris, Herford, Germany, SM1177PT; anti-CD45, Novus Biologicals, Littleton, Colorado, USA, NB1006590APC, anti-CD44, R&D Systems, Minneapolis, Minnesota, USA, MAB5449, anti-CD90, ab225; Abcam, Cambridge, UK). Multipotency of isolated ASCs was tested through osteogenic, chondrogenic, and adipogenic differentiation of cells cultured in StemXVivo kits (R&D System). All abovementioned assays were extensively described previously [21–23].

Isolated cells expressed significant amounts of positive cell surface markers, namely CD90 and CD105, and were negative for CD45 and CD34, which excluded their hematopoietic origin. Moreover, multipotent nature of ASCs was confirmed by positive results of differentiation into osteoblast, chondrocytes, or adipocytes in vitro [20–22].

### Alamar blue viability assay

Cell viability was monitored using the Resazurin-based assay kit (*TOX8*). Briefly, ASCs_EMS_ were plated in 96-well culture plates at 8 × 10^3^cells/well and cultured in 100 μl of DMEM medium. To evaluate the biocompatibility of *C. glomerata* extract with ASCs, cells were treated with 0.5, 1, 1.5, and 2% of *C. glomerata* extract for 72 h. At the end of treatment, all media were removed and 100 μl of a 10% resazurin solution was added to each well. Cells were cultured for an additional 2 h. Afterwards, resazurin metabolization was measured using a spectrometer (Spectrostar Nano; BMG Labtech, Ortenberg, Germany) at the specific wavelengths: 600 nm for resazurin and 690 nm as a background absorbance. The experiment was performed in triplicate.

### Bromodeoxyuridine (BrdU) incorporation assay

DNA synthesis and cell proliferation extend were assessed using the 5-bromo-2-deoxyuridine (BrdU) Cell Proliferation ELISA Kit (Abcam, Cambridge, UK) according to the manufacturer’s instructions. Briefly, ASCs were pre-treated with various concentrations of *C. glomerata* methanolic extract, and then, BrdU was added to cell cultures after 48 h and left overnight at 37 °C to achieve 72 h incubation. Incorporation of BrdU into cellular DNA was determined by labeling fixed cells with anti-BrdU monoclonal antibody, and Goat anti-mouse IgG conjugated with horseradish peroxidase (HRP) as a secondary antibody. HRP substrate degradation was measured with a spectrophotometer plate reader (Spectrostar Nano; BMG Labtech, Ortenberg, Germany) at a wavelength of 450 nm.

### Colony-forming unit-fibroblast (CFU-fs) assay

To evaluate the ability of cells to form colonies, ASCs were seeded in 6-well plates at a density of 100 cells per well in the presence or absence of *C. glomerata* extract at both 0.5% and 1% in appropriate culture medium. Cultures were maintained for 7 days at 37 °C and 5% CO_2_. After fixation in 4% ice-cold paraformaldehyde, cells were stained with pararosaniline solution and colonies of more than 50 cells were observed and counted under an inverted microscope (AxioObserverA1; Zeiss, Oberkochen, Germany). The efficiency of colony forming (*CFU*) was calculated using the following formula:

$$ CFU\_ fs\left(\%\right)=\frac{Number\ Of\ Colonies\ }{Initial\ Cell\ Number} \times 100 $$

### Evaluation of cell morphology

Changes in cellular morphology were monitored using a confocal microscope (Observer Z1 Confocal Spinning Disc V.2 Zeiss with live imaging chamber) as well as a scanning electron microscope (SEM, Zeiss Evo LS 15). Briefly, ASCs_EMS_-treated and control-untreated cells were fixed with 4% paraformaldehyde (PFA) at room temperature for 45 min. Cultures were subsequently washed with HBSS, then cell membranes were permeabilized using 0.1% Triton X-100 solution for 15 min at room temperature. Actin filaments were stained using atto-590-labeled phalloidin (1:800 in HBSS) for 40 min, in the dark at room temperature. Nuclei were labeled by the use of diamidino-2-phenylindole (DAPI), using the ProLong™ Diamond Antifade Mountant with DAPI (Invitrogen™, Poland). Living mitochondria were imaged using the MitoRed fluorescent dye (1:1000 in medium), and incubated for 30 min at 37 °C in a CO_2_ incubator prior to PFA fixation. All images were acquired with a Canon PowerShot camera. Obtained photomicrographs were merged and analyzed using *ImageJ* software (Bethesda, MD, USA). Confocal microscope images were captured as z-stacks having a z-interval of 15, 20, or 25 μm between two consecutive optical slices at a digital size of 512 × 512 pixels.

Detailed cell surface viewing was performed using a scanning electron microscope (SEM; Zeiss EVO LS15) and focused ion beam (FIB; Zeiss, Cobra, AURIGA 60). After fixation of cell cultures in 4% PFA overnight at 4 °C, cells were washed with HBSS three times, dehydrated in graded ethanol mixtures (50–100%), air-dried for 30 min at room temperature, and coated with gold (ScanCoat 6, Oxford). Prepared samples were captured using a SE1 detector at 10 kV filament tension.

### Immunofluorescence staining

EMS and healthy cells were first seeded onto coverslips (Zeiss, Oberkochen, Germany) and pre-treated with *C. glomerata* extract at 0.5% and 1% for 24 h. The remaining medium was after that washed off; cells were rinsed using HBSS and fixed in 4% paraformaldehyde for 40 min at room temperature. Subsequently, the cell membranes were permeabilized with 0.1% Triton X-100 for 15 min at room temperature, washed three times with HBSS, and incubated 45 min in blocking buffer containing 10% Goat Serum and 0.2% Tween-20 in HBSS. Primary antibodies against *Ki-67* (Abcam, Cambridge, UK) (1:500 in HBSS containing 1% Goat Serum and 0.2% Tween-20) were then added to cells and incubated overnight at 4 °C. After washing of antibodies excess, cells were labeled with goat anti-mouse secondary antibodies conjugated with atto-488 (1:1000, Abcam, Cambridge, UK) for 1 h in the dark, at room temperature in a humidified chamber. The immunostained cells were finally mounted in ProLong Gold Antifade containing DAPI (Life Technologies, Warsaw, Poland) and were visualized and imaged using confocal microscope (Zeiss Cell Observer SD).

### Chondrogenic differentiation of equine ASCs

For chondrogenesis experiments, equine ASCs derived from EMS and healthy horses were seeded onto 24-well plates at a density of 2 × 10^4^ per well, or placed into culture tubes at a density of 3 × 10^6^ cells for pellet culture. Regular DMEM culture medium has been afterwards replaced by StemPro™ Chondrogenesis Differentiation Kit (Gibco™, Thermo Fisher Scientific, Poland). Cells were cultured in chondrogenic medium for 10 days in the presence or absence of *C. glomerata* methanolic extract (0.5% and 1%), and culture media were changed every 3 days. At the end of differentiation process, all cultures were subjected to further analysis described below.

### Safranin O straining

The presence of sulfated proteoglycans was detected using the Safranin O staining after 10 days of differentiation. Prior to staining, the chondrogenic micromasses were fixed with 4% paraformaldehyde (PFA) for 15 min at room temperature. Afterwards, cells were washed with HBSS and stained with 0.1% aqueous solution of Safranin O. Stained cells were subsequently observed under an inverted microscope (AxioObserverA1; Zeiss, Oberkochen, Germany), and pictures were taken using a Cannon PowerShot digital camera.

### Apoptosis analysis by flow cytometry

The percentage of chondrogenic EMS and healthy ASCs undergoing apoptosis after chondrogenesis and *C. gomerata* treatment was assessed using the Muse Annexin V & Dead Cell Assay kit™ (Merck Millipore, Darmstadt, Germany) according to the manufacturer’s protocol. All differentiated-treated and differentiated-untreated cells were collected, washed with HBSS, and labeled with the Annexin V & Dead Cell Kit for 20 min at room temperature. The distribution of cells across the four populations: (i) non-apoptotic cells, not undergoing detectable apoptosis: Annexin V (−) and 7-AAD (−); (ii) early apoptotic cells, Annexin V (+) and 7-AAD (−); (iii) late apoptotic cells, Annexin V (+) and 7-AAD (+); (iv) cells that have died through non-apoptotic pathway: Annexin V (−) and 7-AAD (+), was determined by the use of Muse Cell Analyzer (Merck Millipore, Darmstadt, Germany).

### Changes in mitochondrial transmembrane potential determination

Changes in the mitochondrial membrane potential (MMP) were detected on the basis of a MitoPotential lipophilic cationic dye, using the Muse™ MitoPotential Assay kit (Merck Millipore, Darmstadt, Germany). After chondrogenic differentiation in the presence of *C. glomerata* extract, EMS and healthy chondrogenic cells were rinsed with HBSS and stained with the provided fluorescent dyes for 30 min at 37 °C, and the percentage of depolarized cells (depolarized live + depolarized dead) was assessed by the mean of a Muse Cell Analyzer (Merck Millipore, Darmstadt, Germany).

### Intracellular reactive oxygen and nitrogen species analysis

Quantitative measurements of intracellular ROS and RNS, namely Superoxide and Nitric oxide radicals were established using the flow cytometry-based analysis by means of Muse® Oxidative Stress Kit and Muse® Nitric Oxide Kit (Merck Millipore, Darmstadt, Germany) respectively according to the users’ guide instructions. Cells were cultured in chondrogenic differentiation medium with two different concentrations of *C. glomerata* methanolic extract (0.5% and 1%); chondrogenic cells were afterwards washed with HBSS and stained with the corresponding fluorescent dyes for 30 min at 37 °C. Determination of ROS^+^/NO^+^ versus ROS^−^/NO^−^ populations was achieved using a Muse Cell Analyzer (Merck Millipore, Darmstadt, Germany).

### Quantitative real-time reverse transcription polymerase chain reaction (qRT-PCR)

For the detection of chondrogenic-related gene expression, total RNA of *C. glomerata*-treated chondrogenic EMS cells as well as chondrogenic EMS and healthy untreated cells was collected using the TRIzol method according to the manufacturer’s instructions. RNA purity and concentration were measured using a nanospectrophotometer (WPA, Biowave II, Germany). Genomic DNA (gDNA) digestion and cDNA synthesis were performed using a RevertAid First Strand cDNA Synthesis Kit (Thermo Fisher Scientific, Warszawa, Poland) by the mean of a T100 Thermal Cycler (Bio-Rad, Hercules, CA, USA) according to the manufacturer’s instructions.

Expression levels of targeted genes (Table 1) were analyzed by real-time reverse transcription polymerase chain reaction (RT-PCR), using a SensiFAST SYBR Green Kit (Bioline, London, UK) in a CFX Connect™ Real-Time PCR Detection System (Bio-Rad). Reactions performed in a 10-μl volume were subjected to the following cycling conditions: 95 °C for 2 min, followed by 40 cycles at 95 °C for 15 s, annealing for 15 s, and elongation at 72 °C for 15 s. All results were normalized to glyceraldehyde 3-phosphate dehydrogenase (GAPDH) expression. The relative expression level was calculated by comparison of the tested groups with control group using the 2^−ΔΔCQ^ method [29].

**Table 1:** Sequences of primers used in qPCR

Gene	Primer	Sequence 5′–3′	Amplicon length (bp)	Accession no.
*Vimentin*	F:	GCAGGATTTCTCTGCCTCTT	203	XM_014846038.1
	R:	TATTGCTGCACCAAGTGTGT		
*Decorin*	F:	GATGCAGCTAGCCTGAGAGG	248	XM 014841263.1
	R:	GTGTTGTATCCAGGTGGGCA		
*Col2A1*	F:	ATTCCTGGAGCCAAAGGATCTGCT	148	XM_014850051.1
	R:	TGAAGCCAGCAATACCAGGTTCAC		
*ACAN*	F:	TGGTGTCCTCTTCTTGTCGCTTTC	160	XM_014733894.1
	R:	ACGATACATTTGCTGTGCTTCGGC		
*COMP*	F:	AGTGTCGCAAGGATAACTGCGTGA	238	NM_001081856.1
	R:	TCCTGATCTGTGTCCTTCTGGTCA		
*SOX-9*	F:	GAACGCCTTCATGGTGTGGG	225	XM_014736619.1
	R:	TTCTTCACCGACTTCCTCCG		
*Runx-2*	F:	CCAAGTGGCAAGGTTCAACG	165	XM_014837484.1
	R:	TGTCTGTGCCTTCTGGGTTC		
*ALP*	F:	AGGCCAAGATGAGGGTAGCA	352	XM_014852743.1
	R:	TCTCTGGCACTAAGGAGTTGGT		
*VEGF*	F:	CCCACTGCGGAGTTCAACAT	167	XM_014837457.1
	R:	TTTCTCCGCTCTGAGCAAGG		
*CD44*	F:	CCTCCAGCGAAAGAAGCACT	137	NM_001085435.2
	R:	GGCTCGGTCTTTGGTAGTGG		
*p53*	F:	TACTCCCCTGCCCTCAACAA	252	U37120.1
	R:	AGGAATCAGGGCCTTGAGGA		
*Bax*	F:	TTCCGACGGCAACTTCAACT	204	XM_005596728.1
	R:	GGTGACCCAAAGTCGGAGAG		
*Bcl-2*	F:	TTCTTTGAGTTCGGTGGGGT	164	XM_014843802.1
	R:	GGGCCGTACAGTTCCACAA		
*p21*	F:	GAAGAGAAACCCCCAGCTCC	241	XM_003365840.2
	R:	TGACTGCATCAAACCCCACA		
*Sod1 (Cu/Zn SOD)*	F:	CATTCCATCATTGGCCGCAC	130	NW_001867397.1
	R:	GAGCGATCCCAATCACACCA		
*Sod2 (Mn SOD)*	F:	GGACAAACCTGAGCCCCAAT	125	NW_001867408.1
	R:	TTGGACACCAGCCGATACAG		
*CAT*	F:	ACCAAGGTTTGGCCTCACAA	112	XM_014851065.1
	R:	TTGGGTCAAAGGCCAACTGT		
*GAPDH*	F:	GATGCCCCAATGTTTGTGA	250	NM_001163856.1
	R:	AAGCAGGGATGATGTTCTGG		

Sequences: amplicon length and access numbers of the primer sets. *Cal2A1* Collagen type II alpha 1 chain, *ACAN* Aggrecan, *COMP* cartilage oligomeric matrix protein, *Sox-9* transcription factor SOX-9, *Runx-2* Runt-related transcription factor 2, *ALP* alkaline phosphatase, *VEGF* vascular endothelial growth factor, *CD44* Homing cell adhesion molecule, *p21* cyclin dependent kinase inhibitor 1A, *p53* tumor suppressor p53, *BCL-2* B-cell lymphoma 2, *BAX* BCL-2-associated X protein, *Sod1 (Cu/Zn SOD)* copper-zinc-dependant superoxide dismutase, *(CuZnSOD*; *Sod2 (Mn SOD)* manganese-dependent superoxide dismutase (MnSOD), *GADPH* glyceraldehyde-3-phosphate dehydrogenase

### miRNA expression analysis

Total RNA was used to generate cDNA by means of a Mir-X miRNA First-Strand Synthesis RT Kit (Clontech Laboratories, Inc., Mountain View, CA, USA) according to the manufacturer’s instructions. The cycle parameters for the RT reaction were 30 °C for 10 min, 55 °C for 5 min, 37 °C for 1 h, and 85 °C for 5 min. Subsequently, the synthesized cDNA was used for real-time quantitative PCR with SensiFAST SYBR Green Kit (Bioline, London, UK). The reaction was performed using 0.5 μL of template, and the final concentration of primers was 0.4 μM. The following cycling conditions were applied during the reaction: a temperature of 95 °C for 10 s, followed by 35 cycles at a temperature of 95 °C for 5 s and annealing temperature of 58.8 °C for 20 s with a single fluorescence measurement. The list of miR-specific primers used in the reaction is presented in Table 2. The mRQ 3′ primer and U6snRNA primers were provided with the RT kit. The average fold change in the gene expression of experimental cultures was compared with control cultures and calculated by the 2^−ΔΔCQ^ method in relation to U6snRNA.

**Table 2:** Sequences of primers used for detection of microRNA

Gene	Primer sequence 5′–3′	Accession no.
*miR-27b*	AGAGCUUAGCUGAUUGGUGAAC	MIMAT0004588
*miR-34a*	UGGCAGUGUCUUAGCUGGUUGU	MIMAT0000255
*miR-140-3p*	TACCACAGGGTAGAACCACGGA	MIMAT0004597
*miR-145-5p*	GTCCAGTTTTCCCAGGAATCCCT	MIMAT0000437
*miR-146a-5p*	TGAGAACTGAATTCCATGGGTT	MIMAT0000449
*miR-449a*	UGGCAGUGUAUUGUUAGCUGGU	MIMAT0001541

### Statistical analysis

Statistical analysis was performed using GraphPad Prism 5.0 (San Diego, CA, USA). Statistical significance was determined using one-way analysis of variance (ANOVA) with Dunett’s post hoc multiple comparison test. Asterisk (*) and Hash (#) signs indicated statistical significance in EqASCs_EMS_ control or *C. glomerata*-treated groups versus healthy control or EqASCs_Healthy_ control versus *C. glomerata*-treated groups, respectively. *p* values lower than 0.05 (*p < 0.05*) were summarized with one asterisk/hash (*/#), *p < 0.01* with two asterisks/hashes (**/##), and *p < 0.001* with three asterisks/hashes (***/###).

## Results

### *Cladophora glomerata* biocompatibility with EqASCs

**Fig. 1:**
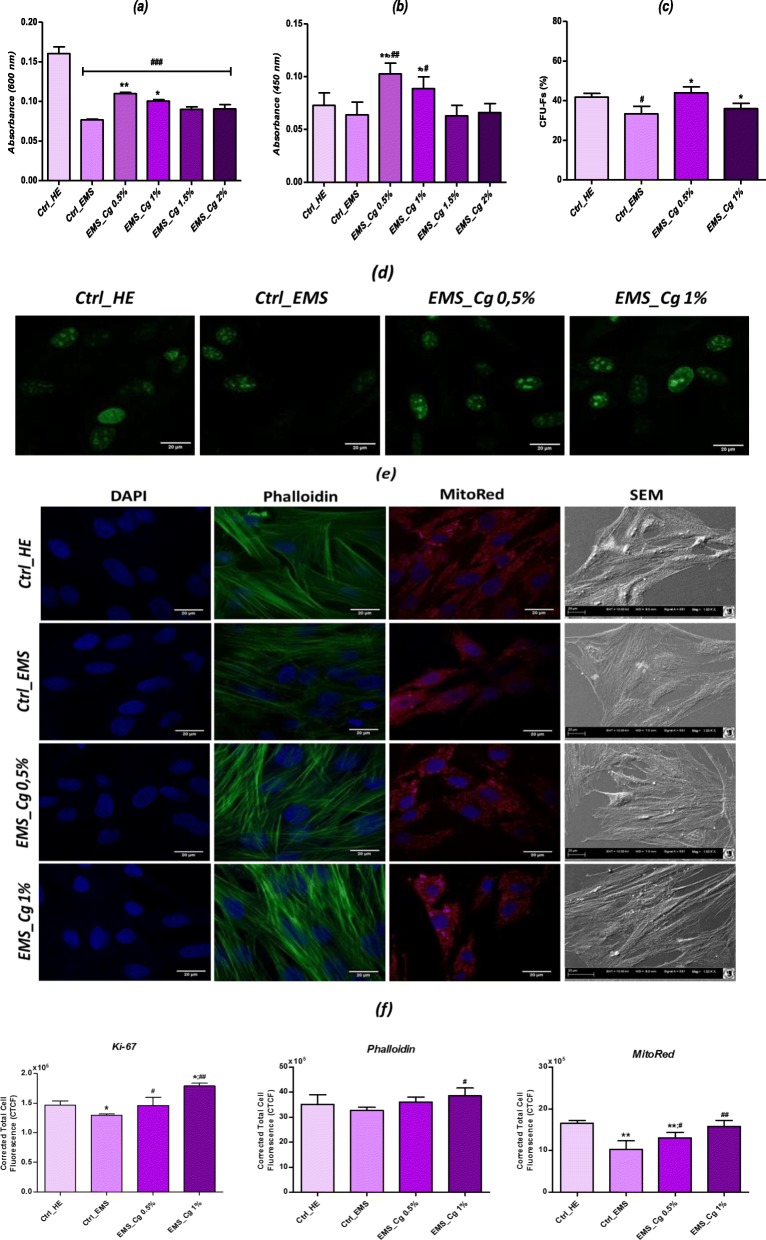
Influence of *C. glomerata* extract on EqASC condition. **a** Histograms represent the average absorbance at 600 nm of the metabolized rezasurin dye by treated and untreated living cells. **b** Bar charts represent the mean of the absorbance of the BrdU level incorporated in the neo-synthesized DNA of the treated and untreated cells. **c** Clonogenic fibroblast precursor (CFU-F) assay for treated and untreated ESCs. **d** Representative photomicrographs for Ki-67 immunostaining after extract treatment. **e** DAPI, MitoRed, and Phalloidin labeled cells were observed using an inverted epi-fluorescent confocal microscope; scale bar size 20 μm; magnification was set at 60-fold. Cell surface and shape was assessed by the mean of scanning electron microscope; scale bar size 30 μm; images were acquired under 1000-fold magnification. **f** Bar charts representation of corrected total cell fluorescence (CTCF) for Ki-67, Phalloidin and MitoRed fluorescent staining. The results are expressed as the mean of 3 different experiments ± SD. Asterisk (*) refers to comparison of treated groups to untreated EMS cells. Hashtag (#) refers to comparison of treated groups to untreated healthy cells. *^/#^*p <* 0.05, **^/##^*p <* 0.01, ***/^*###*^*p* < 0.001

The effects of *C. glomerata* extract on EqASC condition were investigated in terms of viability, proliferation, and cellular morphology (Fig. 1).

Resazurin-based assay (TOX-8) showed that ASCs_EMS_ exhibited significant reduced number of living cells as compared to control healthy cells (*p* < 0.001). Moreover, obtained results indicated that algal extract did not affect negatively ASC viability and metabolic activity in a range of concentrations of 0.5 to 2%; interestingly, treatment with both 0.5% and 1% extract significantly promoted ASCs_EMS_ viability (Fig. 1a) in comparison to the EMS-untreated cell group (*p* < 0.01; *p <* 0.05). The proliferative effect of the *C. glomerata* extract was examined using the BrdU incorporation method. As shown in Fig. 1b, there were markedly more BrdU-labeled cells in both 0.5% and 1% *C. glomerata* extract-treated cells *(p* < 0.01; *p* < 0.05), relative to increased level of newly synthesized DNA, as compared to healthy and EMS control groups, evidencing a stimulatory effect of the extract on ASC proliferation. Moreover, the cell-cycle-associated Ki-67 protein has been stained in treated and untreated cells as marker of proliferative cells. Cultures supplemented with *C. glomerata* methanolic extract exhibited highly green-related fluorescence emanating from the cellular nuclei when observed under a confocal microscope, confirming the proliferative potential of the extract (Fig. 1d). Influence of tested extract on the ability of ASCs to form colonies was also examined via the clonogenic fibroblast precursor (CFU-F) assay (Fig. 1c). The number of colonies consisted of more than 50 cells was decreased in EMS cells compared to the number of colonies formed by non-diseased cells (*p* < 0.05). Through this test, it was also found that macroalgal extract greatly enhanced the ability of single EMS cells to organize into colonies, since no statistical differences were observed when compared to healthy phenotype. General morphological features of treated and untreated cells were also assessed. Observations with scanning electron microscope pointed out that cells conditioned with *C. glomerata* extract displayed more fibroblast-like, elongated morphology and greatest net of cytoskeletal projections which connected adjacent cells, whereas EMS-untreated cells showed rather flat and no longer of bipolar shape (Fig. 1e). Confocal microscopy evidenced a larger number of oval nuclei in all EMS-treated cells as well as healthy cells compared to untreated EMS cells that showed a number of strands of DAPI-stained nuclei, some of which were irregularly shaped and relatively enlarged; moreover, one or more prominent nucleoli were observed in the nucleus of both untreated and treated cells. Phalloidin staining showed a relatively well structured and dense cytoskeleton network for *C. glomerata*-treated cells, comparable to that of the healthy cells; actin filaments were homogeneously distributed and constitute a uniform and continuous spindle. Mitochondria imaging highlighted abundant fluorescent red spots in the cytoplasm of treated cells corresponding to the metabolically active mitochondria, by contrast to the mitochondrial networks of lower fluorescent intensity observed in EMS control cells (Fig. 1e).

### Morphological features of chondrogenic cells

**Fig. 2:**
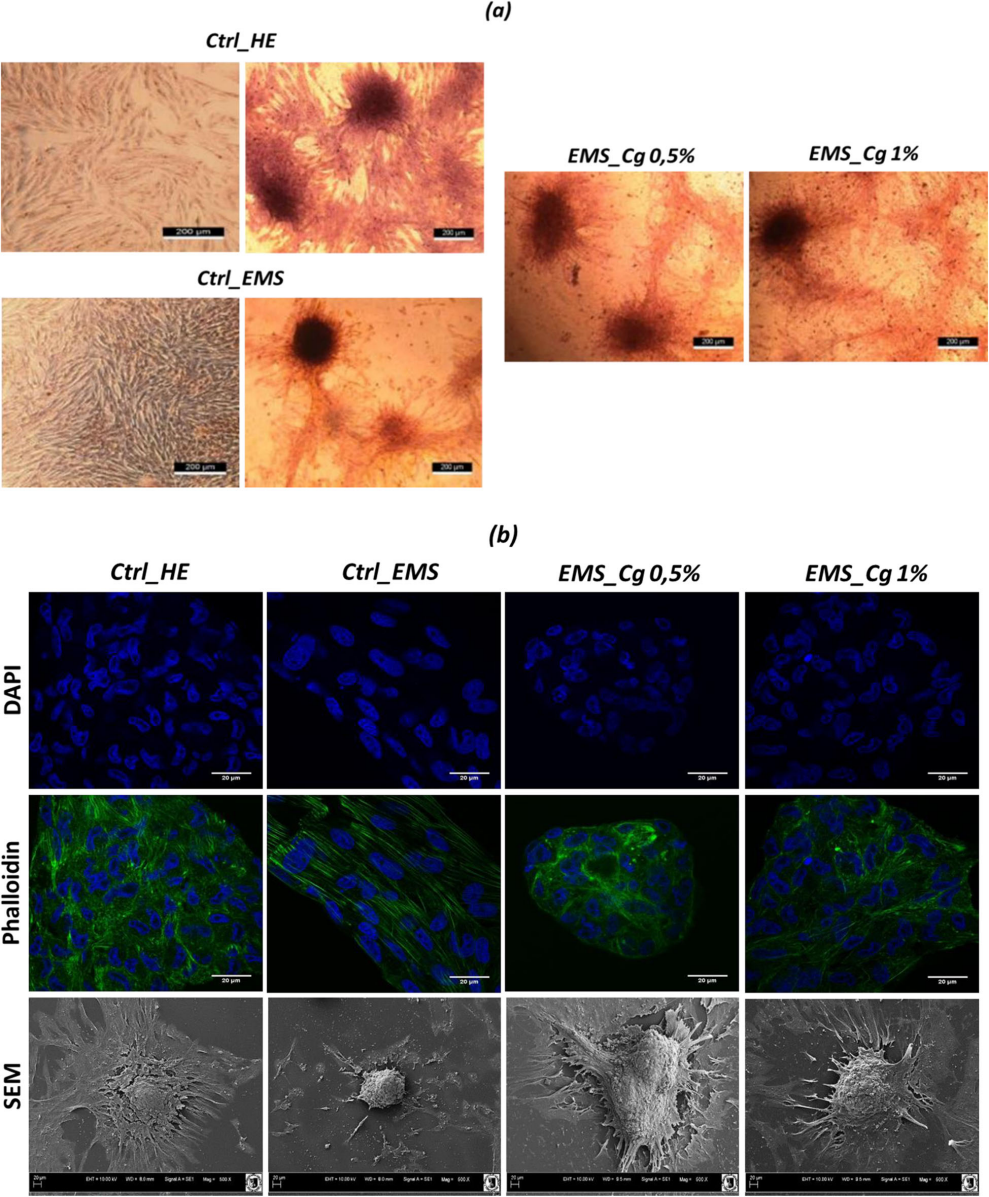
Evaluation of chondrogenic cells morphology. **a** Chondrogenic cultures were stained with Safranin O and observed under an inverted microscope, bar size 200 μm; magnification was set at 40-fold. **b** DAPI and Phalloidin labeled cells were observed using an inverted epi-fluorescent confocal microscope; scale bar size 20 μm; magnification was set at 60-fold. Cell surface and shape was assessed by the mean of scanning electron microscope; scale bar size 30 μm; images were acquired under 1000-fold magnification

Influence of *Cladophora glomerata* methanolic extract on chondrogenic differentiation ability of EqASCs_EMS_ was established in comparison to the EqASCs_EMS_ as well as EqASCs_healthy_ cultured in chondrogenic and non-chondrogenic culture conditions. The Safranin O staining of extracellular matrix in cultures (Fig. 2a) showed the total absence of chondrogenic nodules in non-differentiated cultures of ASCs whether it is EMS or healthy cells, whereas ASC cultures supplemented with algal extract were characterized by the prominent formation of glycosaminoglycan-rich nodules under chondrogenic conditions after 10 days of differentiation. Cells changed their morphology from adherent monolayer spindle-shaped cells to layered nodule-like cell clusters. SEM images evidenced many chondrogenic nodule-like structures that tended to form connections among each other with many extracellular matrix vesicles, which appeared more defined in extract-treated ASCs_EMS_ compared to EMS-untreated cells. Developmental changes in the architecture of cartilage nodules during differentiation were monitored using confocal laser scanning microscopy. DAPI staining revealed distinct cell morphology arranging as aggregates whose contour of clusters appeared as slightly rugged spheroid. Chondrocytes in monolayer culture derived from ASCs_EMS_-treated cells rapidly developed a rounded cell shape with extensive stress fiber reorganization of the actin cytoskeleton to a cortical pattern parallel to the cells rounding, suggesting that *C. glomerata* methanolic extract enhanced the establishment of a chondrocyte-specific cell shape and actin organization (Fig. 2b).

### Chondrogenesis marker gene expression

**Fig. 3:**
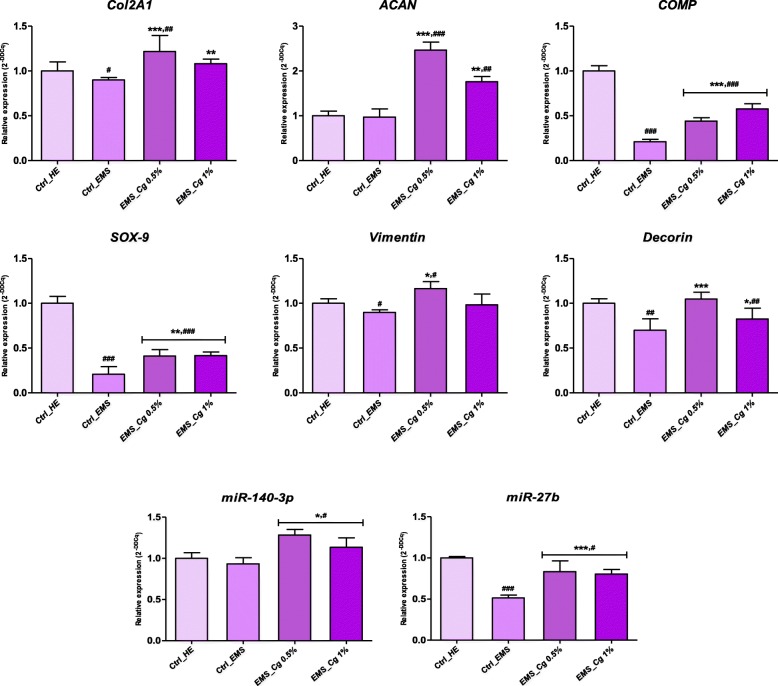
Relative mRNA expression analysis of the chondrogenic marker genes *collagen type II*, *Aggrecan*, *Sox9*, *Vimentin*, *Decorin*, and *COMP* as well as the regulatory *miR-140-3p* and *miR-27b* in the *C. glomerata*-treated and *C. glomerata*-untreated EqASCs_EMS_ during in vitro-induced chondrogenesis. The results are expressed as the mean of 3 different experiments ± SD. Asterisk (*) refers to comparison of treated groups to untreated EMS cells. Hashtag (#) refers to comparison of treated groups to untreated healthy cells. *^/#^*p* < 0.05, **^/##^*p* < 0.01, ***/^*###*^*p <* 0.001

The impact of the *C. glomerata* extract on the maintenance of cartilage matrix biosynthetic activity in differentiated cells was assessed on the gene expression of cartilage macromolecules, as well as the key chondrocyte transcription factors, namely aggrecan, collagen, and cartilage oligomeric matrix protein, *Vimentin*, *Decorin*, and *Sox9*. qRT-PCR analysis (Fig. 3) revealed that chondrogenic cells derived from ASCs_EMS_ showed significantly lower expression of all key chondrogenesis markers compared to differentiated healthy ASCs (*p <* 0.001), demonstrating an impaired chondrogenic potential in EqASCs_EMS_. In response to 0.5% of *C. glomerata* extract, all collagen-related genes, namely *Col2A1*, *COMP*, and *Vimentin* gene expression, were significantly unregulated as compared to EMS-untreated cells (*p* < 0.001). Indeed, chondrogenic cells supplemented with 0.5% extract exhibited around 1.3, 2, and 1.45-fold higher expression for *Col2A1*, *COMP*, and *Vimentin* respectively in opposition to EMS control cells (Fig. 3). Chondrogenic cultures supplemented with 1% algal extract also showed a significant stimulation of the expression of the same genes; however, no correlation between the concentration increasing and the obtained effect was observed, indicating that the activity of methanolic extract does not appear to be dose-dependent. Assessment of proteoglycan-related gene expression showed significant upregulation of *ACAN* and *Decorin* after 10 days differentiation in the presence of both 0.5% and 1% concentrations of *C. glomerata* extract. Subsequently, the expression of *Sox9* transcript, which was markedly reduced during the metabolic condition (*p* < 0.001), was partially restored (*p* < 0.01) in cultures supplemented with the macroalgae methanolic extract, thus potentiating the chondrogenic differentiation process of EqASCs_EMS_ (Fig. 3). Similar tendency was recorded regarding expression of chondrogenic-specific *miR-140-3p* and *miR-27b* that are well known cartilage matrix degradation repressors and regulators of chondrocyte homeostasis (Fig. 3). *C. glomerata* extract noticeably induced targeted microRNA expression in ASCs_EMS_-derived chondrogenic cells when compared to untreated group of cells (*p* < 0.05; *p* < 0.001).

### Apoptosis evaluation during chondrogenesis

**Fig. 4:**
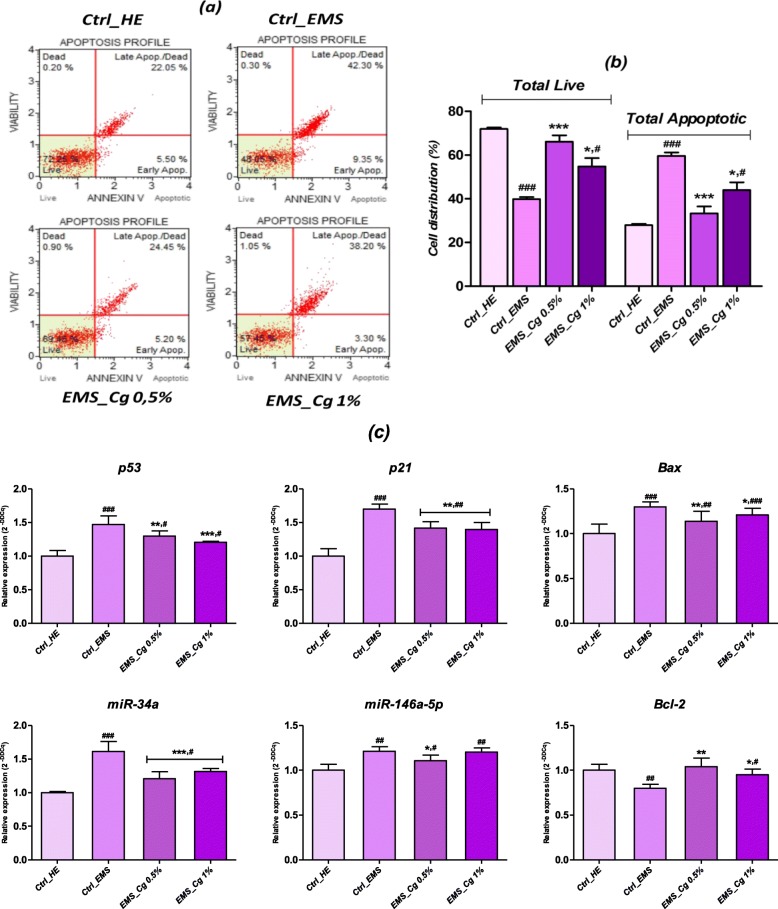
Assessment of apoptosis in ASCs_EMS_-derived chondrogenic cells. Cells were assayed with Annexin V/PI staining to measure the percentage of viable cells (Annexin V−/PI−), early apoptotic cells (Annexin V+/PI−), late apoptotic cells (Annexin V+/PI+), and necrotic cells (Annexin V−/PI+). **a** Apoptosis profile plots. Each plot is a representative figure of the three replicates of each determination. **b** Bar charts depicting percentage of live and total apoptotic cells. **c** Representative bar charts of relative expression of apoptotic key markers. Representative data from three independent experiments are shown± SD (*n* = 3 Asterisk (*) refers to comparison of treated groups to untreated EMS cells. Hashtag (#) refers to comparison of treated groups to untreated healthy cells. *^/#^*p* < 0.05, **^/##^*p <* 0.01, ***/^*###*^*p <* 0.001

To examine whether the exposure to *C. glomerata* methanolic extract affects the ASCs_EMS_-associated apoptosis during chondrogenesis process, ASC chondrogenic pellets were subjected to apoptotic assays. Annexin V/7-AAD staining of ASCs_EMS_ recovered from the pellets revealed that the apoptotic rates on day 11 of chondrogenesis were 59.63 ± 1.6% and 27.86 ± 0.63% for EMS and healthy cultures respectively (Fig. 4b). The addition of the two different concentrations of the methanolic extract throughout the chondrogenesis period has significantly reduced the rate of apoptotic cells of the order of 33.33 ± 0.56% and 43.93 ± 1.03 respectively. These data suggest that *Cladophora* extract suppressed EMS chondrogenesis-induced apoptosis in ASCs.

Changes in gene expression of main related markers involved in apoptotic pathway regulation were monitored as well, after complete chondrogenesis in all tested groups through the analysis of anti-apoptotic and pro-apoptotic gene transcription. Figure 4 c shows that differentiated ASCs_EMS_ exhibited significant higher expression of main pro-apoptotic genes, namely *p53*, *p21*, and *Bax* as well as microRNAs implicated in chondrocyte apoptosis promoting *miR-34a* and *miR-146a-5p*, when compared to normal untreated ASCs (*p* < 0.01; *p* < 0.001). Moreover, the same group was characterized by highly reduced *Bcl-2* mRNA as compared to healthy phenotype (*p <* 0.01). RT-qPCR results (Fig. 4c) confirmed the anti-apoptotic properties of *C. glomerata* methanolic extract on chondrogenic EMS cells; in fact, exposure of EMS cell cultures throughout the chondrogenesis process to the two concentrations of algal extract (0.5% and 1%l) markedly reduced expression of pro-apoptotic *p53* and *p21* genes, and *miR-146a-5p* and *miR-34a* apoptosis promoter factors as well, suggesting a possible restraint of that apoptosis pathway (*p <* 0.05; *p* < 0.01; *p <* 0.001). Furthermore, algal methanolic extract significantly promoted chondrogenic EMS cells survival by modulating and restoring the transcription of the *Bcl-2*-survival gene and reducing the level of *Bax* transcript (*p <* 0.001).

### Measurement of oxidative stress level

**Fig. 5:**
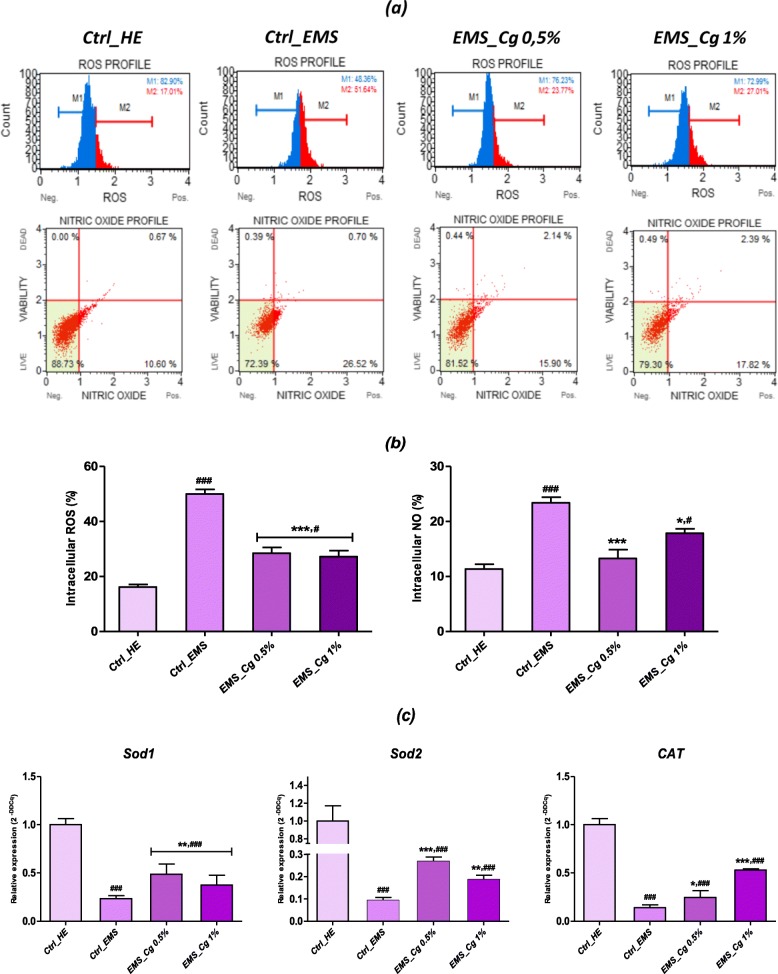
*C. glomerata* reduces oxidative stress during ASCs_EMS_ chondrogenesis. **a** Representative plots depicting cell stained with DHE and DAX-J2 Orange dyes and evaluated using a flow cytometer. **b** Each bar summarizes the mean percentage ± SD of three independent experiments for total intracellular ROS- and NO-positive cells. **c** Relative gene expression of main endogenous antioxidant enzymes. Asterisk (*) refers to comparison of treated groups to untreated EMS cells. Hashtag (#) refers to comparison of treated groups to untreated healthy cells. *^/#^*p <* 0.05, **^/##^*p* < 0.01, ***/^*###*^*p* < 0.001

To examine the role of *C. glomerata* extract on oxidative stress mediating cell death during ASCs_EMS_ chondrogenesis, intracellular ROS and NO were quantified in chondrogenic cell micromasses (Fig. 5). A significant increase in intracellular ROS and NO was found in chondrogenic cultures derived from EMS-untreated cells as compared to healthy control group (*p* < 0.001). However, groups treated with both concentrations of *C. glomerata* extract showed significant decreases in intracellular ROS and NO (*p <* 0.001) from 50.01 ± 1.64% to 23.4 ± 1.02% respectively in EMS untreated control group, to 28.46 ± 1.12% and 27.22 ± 0.97% of ROS, as well as 13.29 ± 1.3% and 17.86 ± 0.84% of NO, for groups treated with 0.5% and 1% extract respectively (Fig. 5b). Possible modulatory effects of *C. glomerata* extract on gene expression of main antioxidant enzymes were established using qRT-PCR analysis (Fig. 5c). EMS-untreated cells were markedly impaired in terms of endogenous antioxidant defenses, as evidenced by the downregulation of *Sod1*, *Sod2*, and *CAT* transcripts as opposed to healthy control cells (*p* < 0.001). The simultaneous application of algae extracts during chondrogenesis of EMS cells strongly modulated the transcription of the genes encoding for the main antioxidant enzymes, catalase and superoxide dismutase when compared to untreated EMS cells (*p* < 0.001; *p* < 0.05), suggesting that the reduction of free radical rates within the cells operated by the extract would be partly associated with a reinforcement of own cell antioxidant defenses.

### Mitochondrial membrane depolarization

**Fig. 6:**
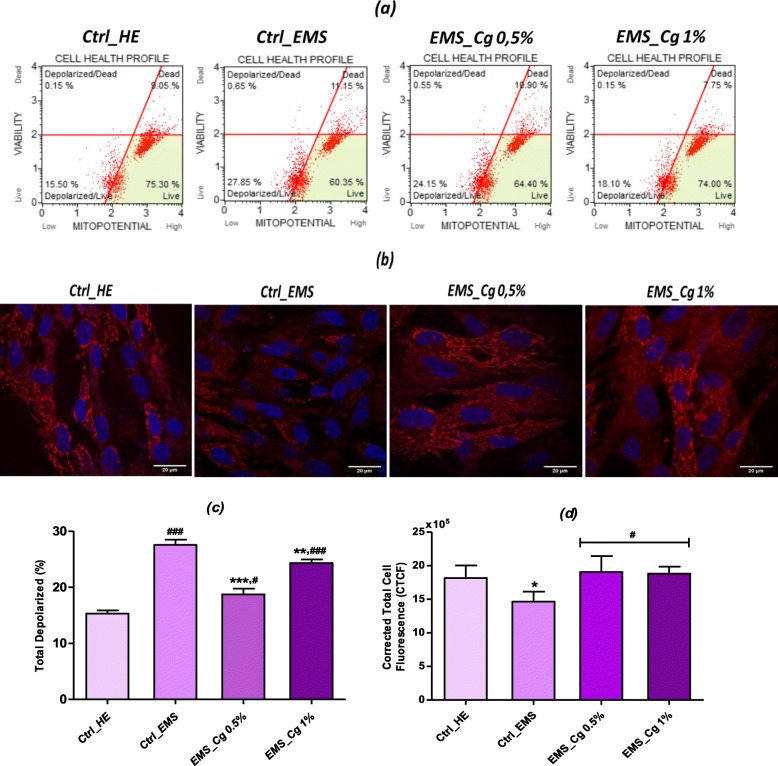
Mitochondrial membrane potential analysis. **a** Scattered blots representation of live and dead depolarized cells percentages for one representative experiment. **b** MitoRed stained cells were observed using an inverted epi-fluorescent confocal microscope; scale bar size 20 μm; magnification was set at 60-fold. **c** Bar charts represent the average percentages ± SD of total depolarization for three repetitions. **d** Bar chart representation of corrected total cell fluorescence (CTCF) for MitoRed fluorescent staining. Asterisk (*) refers to comparison of treated groups to untreated EMS cells. Hashtag (#) refers to comparison of treated groups to untreated healthy cells. *^/#^*p* < 0.05, **^/##^*p* < 0.01, ***/^*###*^*p <* 0.001

The effects on depolarization of the mitochondrial membranes in chondrogenic cells derived from both ASCs_EMS_ and healthy cells (loss of ΔΨm) were measured by Muse system when treated with the two concentrations of *C. glomerata* extract (Fig. 6). In EMS-untreated control group, cells exhibited an important increase of the mean percentage of cellular depolarization when compared to healthy phenotype (*p* < 0.001). A significant change in the ΔΨm was evident in both 0.5% and 1% extract-treated cells in opposition to the non-treated group (*p <* 0.001); tested extract stimulated mitochondrial membrane potentiation reflected by decrease in percentage of depolarized cells from 27.58 ± 0.95% in control EMS group to 18.76 ± 0.99% and 24.31 ± 0.66% for extract at both concentrations respectively (Fig. 6c).

Since mitochondrial dysfunction is a reliable indicator of cellular health, confocal imaging of MitoRed-stained mitochondria was performed (Fig. 6b; Fig. 6d). Representative confocal micrographs of ASCs_EMS_ loaded with MitoRed after chondrogenic differentiation showed marked decrease in dye-red fluorescence in comparison to healthy group, which is associated to MS-induced mitochondrial potential collapse. The supplementation of chondrogenic medium with *C. glomerata* methanolic extract resulted in great improvement of mitochondrial condition, which displayed strong fluorescent signals unlike the control group. Stained mitochondria were abundantly present and relatively uniformly distributed in the cytoplasm, forming a continuous well-defined network.

### Cartilage hypertrophy marker gene expression

**Fig. 7:**
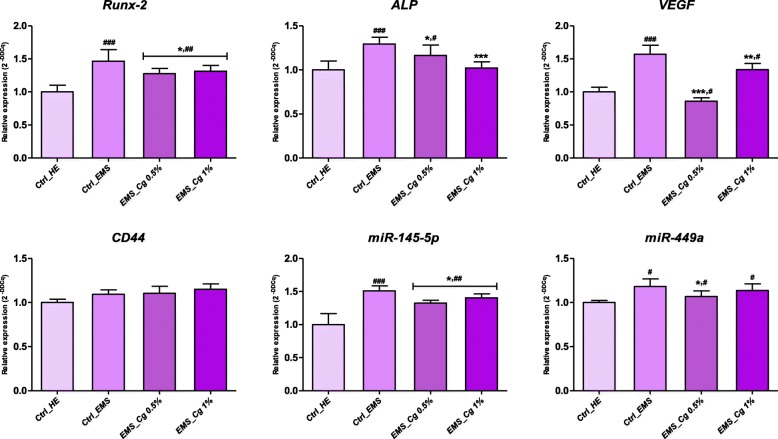
Influence of *C. glomerata* extract on hypertrophy-related gene transcripts *Runx2*, *ALP*, *VEGF*, *CD44*, and both *miR-144-5p* and *miR-449a*. Histograms refer to average relative expression normalized du GAPDH housekeeping gene. Asterisk (*) refers to comparison of treated groups to untreated EMS cells. Hashtag (#) refers to comparison of treated groups to untreated healthy cells. *^/#^*p <* 0.05, **^/##^*p* < 0.01, ***/^*###*^*p* < 0.001

*C. glomerata* extract was examined whether it inhibits hypertrophic differentiation of chondrocytes by performing pellet culture of ASCs_EMS_ in the presence or absence of extract. Recorded data (Fig. 7) clearly showed gene overexpression of the main factors initiating chondrocyte hypertrophy, in differentiated cells derived from EMS cultures compared to healthy control cultures (*p* < 0.001). The addition of 0.5% and 1% of algae extract significantly decreased the expression levels of *Runx2*, *ALP*, and *VEGF* up to 0.9, 1.27, and 1.8-folds respectively relative to untreated EMS group; results suggest that *C. glomerata* extract could exhibit the potential to suppress hypertrophy during chondrogenesis.

Screening of potential effects of *C. glomerata* extract on miRNAs involved in ASC chondrogenic defects was also undertaken. Real-time PCR showed that both *miR-145-5p* and *miR-449a* miRNAs that are closely related to cartilage degeneration were significantly upregulated in ASCs_EMS_ after cartilage induction in reference to healthy cells (*p <* 0.05; *p* < 0.01). Stimulation of EMS cells during chondrogenesis with *C. glomerata* extract affected noticeably the transcription of the targeted microRNAs compared to untreated control cells (*p <* 0.05).

## Discussion

Being able to differentiate into various cell lineages, MSCs represent a promising option for the therapeutic management of degenerative pathologies affecting the muscular skeletal system, such as chondral lesions or osteoarthritis; on the other hand, it is now accepted that these same cells may be affected by certain existing disorders, which may impair their various physiological activities [30]. In this study, we report that using in vitro micromass culture models of chondrogenesis established from ASCs_EMS_, the *Cladophora glomerata* methanolic extract was able to significantly enhance differentiation by promoting cell viability and upregulating a number of genes associated with chondrogenesis that was reflected in chondrogenic cell morphology. Moreover, apoptosis and hypertrophic factors were downregulated as well. Similarly, expression of microRNAs involved in the process was differentially regulated by the extract. In this work, we showed that different concentrations of *C. glomerata* methanolic extract promote cell proliferation and morphology when ASCs_EMS_ are not undergoing inductive differentiation, according to the obtained cell metabolic activity and proliferation data and micrographs as well. Comparison of cell proliferation established from BrdU labeling and Ki67 immunofluorescence staining under supplementation with 0.5% and 1% *C. glomerata* extract revealed a significant higher growth rate than in cultures without extract. These results are in concordance with previous studies, which demonstrated that *C. glomerata* macroalgae is able to improve EqASCs_EMS_ condition in terms of viability, morphology, apoptosis, oxidative stress, and mitochondrial activity [19]. Subsequently, we further investigated the influence of algal extract on the chondrogenic differentiation of these cells. Accordingly, we aimed to optimize the chondrogenic culture conditions for the in vitro induction of ASCs_EMS_ by testing two different concentrations of *Cladophora* in basic chondrogenic-inductive medium. Our results regarding cell morphology in basic chondrogenic-inductive medium containing 0.5% and 1% extract revealed a markedly improved cellular architectural organization. Safranin O staining of extracellular matrix in cultures showed visible formation of glycosaminoglycan-rich nodules under chondrogenic conditions after 10 days of differentiation. Chondrogenesis of EqASCs_EMS_ cultured in the presence of *C. glomerata* extract was also suggested by similar chondrogenic-like nodule morphology, a tendency to form inter-chondrogenic nodule connections with numerous fibers formed by the cells, which suggest possible formation of collagen; moreover, many extracellular matrix vesicles were present and abundant at the surface of micromasses. The RT-PCR data are in general agreement with the morphological observations, showing high levels of chondrogenic mRNAs in aggregates after methanolic algal extract stimulation, such as *ACAN*, *COMP*, *Col2A1*, *Sox9*, *DCR*, and *Vimentin*. The *Sox9* transcription factor is considered as an essential component in the development and maturation of cartilage tissue. As one of the first condensing chondrocytes marker, its expression begins with the multipotent skeletal progenitor stage and is maintained throughout life in mature chondrocytes of healthy articular cartilage and underlies chondrocyte differentiation through transcriptional activation of many genes that are essential to build and maintain health cartilage [31]. *Sox9* controls mesenchymal condensation as well as chondrogenic lineage commitment and differentiation by inducing many other targets such as *Sox5* and *Sox6* expression. Subsequently, these factors cooperate as a pivotal complex for activating the transcription of chondrocyte-specific genes, including typical cartilage ECM genes *Col2a1*, *Col9a1*, *Col11a2*, *ACAN*, *CD-rap*, and others like *COMP* [32, 33]. Type II collagen and aggrecan are the principal and specific matrix components in cartilage that are produced by chondrocytes, while cartilage oligomeric matrix protein (COMP) plays an important role in cartilage cell-matrix interactions [34, 35]. In this study, we observed that ASCs affected by metabolic syndrome exhibited a significant reduction in their chondrogenic potency as characterized by a collapse of the *Sox9* gene transcription and a consequent downregulation of the related cartilaginous factors. Whereas pro-inflammatory and anti-inflammatory cytokines are believed to play a crucial role in ASC cell differentiation processes [36], due to the MS-associated dysregulation of ASCs and uncontrolled inflammatory microenvironment, it has been shown that Interleukin-1 (IL-1) and tumor necrosis factor alpha (TNF-α) can exert an inhibitory effect on *Sox9* expression, thus interfering with the underlying chondrogenic signaling pathways [37]. Our previous data already reported on the regulatory effect of *C. glomerata* methanolic extract regarding pro-inflammatory cytokines IL-1, IL-6, and TNF-α produced by EqASCs under oxidative stress and inflammatory conditions; indeed, the extract was able to significantly downregulate the pro-inflammatory transcripts and enhance in the same time the expression of IL-10 anti-inflammatory cytokine [27]. As a result, the observed modulatory effect of *Cladophora* on the expression of *Sox9* factor and consequently of other chondrogenic markers could be partly related to the inflammatory profile mitigation in ASCs_EMS_. On the other hand, analysis of microRNAs involved in chondrogenesis regulation indicated that extract at both concentrations (i.e., 0.5% and 1%) markedly upregulated expression of *miR140-3p* and *miR27b* in opposition of EMS-untreated cells that exhibited impaired regulatory microRNA transcription. The level of *miR-140*, which is highly expressed in chondrocytes, is known to increase during chondrogenic differentiation from MSCs. This prominent microRNA can target osteoarthritis (OA)-related mRNAs, *ADAMTS-5*, *MMP13*, *COl2a1*, and *ACAN* in human articular chondrocytes. Moreover, it has been shown that *miR140* interacts with the *PTHrP-HDAC4* pathway indirectly by regulating the expression of myocyte enhancer factor 2C to control chondrocyte differentiation as well [38, 39]. *miR27b* is, for its part, largely involved in the repression and inhibition of *MMP13*, a key enzyme involved in the degradation of the cartilaginous matrix; other study highlighted also that *miR-27b* can modulate *Sox9* and *Col2a1* expression at both mRNA and protein levels [40]. Although hypertrophy is an important physiological process for the formation of bone matrix on the partially degraded cartilage, inappropriate hypertrophic chondrocytes are associated with the onset and progression of osteoarthritis, ECM mineralization, increased vascularity, and apoptosis of chondrocytes [41]. In this investigation, an early onset of expression of chondrocyte hypertrophy-associated genes, including *Runx2*, *ALP*, and *VEGF*, has been evidenced in chondrogenic cells derived from ASCs_EMS_. Further overexpression of some microRNAs namely, *miR145-5p* and *miR449a*, that are closely related to cartilage degeneration was also recorded for this group. Chondrocyte hypertrophy is known to be controlled by a complex regulatory network consisting of different markers; among them, *Runx2* is a key factor whose overexpression has been largely implicated in the progression of degenerative cartilage affections, such as osteoarthritis and bone remodeling defects. The latter mediates and controls cartilage degradation via matrix metalloproteinase-13 (*MMP-13*) through its induction by *HIF-2α*. Consecutively, *Collagen X*, *MMP-13*, and *ALP* were identified as hypertrophic markers and presented overexpression during hypertrophic differentiation [42]. The activation of *Runx2* through the signaling pathways of hypertrophy, notably involving *TNT*, *BMP*, and *IHH*, triggers the transcription of *collagen X*, *MMP-13*, *ALP*, and *VEGF*, which initiate the process of hypertrophy, calcification, and apoptosis [41, 43]. *miR-145* is recognized as a key mediator of cartilage degeneration that can antagonize early chondrogenic differentiation via attenuating the expression of chondrogenic markers, including *Sox9*, *Col2A1*, *Agc1*, *COMP*, *Col9A2*, and *Col11A* at the posttranscriptional level when overexpressed [44]. *miR-449a* also belongs to the microRNA-negative regulators family. It mainly suppresses expression of *Lef1*, a critical component of the canonical *Wnt* signaling pathway. This suppression subsequently downregulates *Col2A1* and *Sox9* expression and reduces proteoglycan production [45]. The addition of the two different concentrations of *C. glomerata* extract to the chondrogenic culture medium of ASCs_EMS_ resulted in a strong regulation of hypertrophy by repressing expression of the main factors involved in the process at the mRNA level. In addition, a simultaneous attenuation of *miR-146a-5p* and *miR-449a* expression was observed, suggesting that macroalgal extract could also positively regulates *Sox9* and cartilage-related gene expression, and thus promotes chondrogenic differentiation through pathological hypertrophy as well as ECM degradation alleviation. As a polyphenolic-rich extract, many studies reported on the potent anti-hypertrophic potential of different phenolic compounds, these later were manly showed to act as *Runx2* regulators and inhibitors of *collagen II* and aggrecan-destroying *MMP-13*. Moreover, the reduction of inflammatory cytokines such as *IL-1β* that induces glycosaminoglycan release from cartilage, *TNF-α* that induce *MMP-1*, *MMP-3*, *ERK* (extracellular signal-regulated kinase), *p38*, *JNK* and *AP-1* activities, and *NF-κB* and *Cox-2* pathways by polyphenols has been proposed as a possible mechanism of cartilage degeneration and hypertrophy attenuation [46–49]. Since hypertrophic chondrocytes inevitably undergo programmed cell death, apoptosis can be considered as a reliable marker for cartilage destruction [50]. Prominent apoptotic profile was obvious in EMS chondrogenic cultures after 10 days differentiation; expression levels of all *p53*, *p21*, *Bax*, *miR-146a-5p*, and *miR-34a* pro-apoptotic factors have been highlighted after RT-PCR assessment; a large population of apoptotic cells was found for the same group contrasted by a reduced cell viability rate as well. Basically, chondrocyte viability is an essential issue for maintaining the integrity and homeostasis of articular cartilage; however, reduced cellularity (due to necrosis or apoptosis) can trigger matrix degeneration and be associated with onset and progression of cartilage loss integrity [30]. *VEGF* is a crucial mediator in vascularization and removal of terminal hypertrophic chondrocytes during embryonic bone development, while it is also an essential coordinator of chondrocyte death [51]. On the other hand, *miR-146a* was found to play a role in the damage of chondrocytes due to mechanical injury [52]. Taken together, recent study demonstrated that increase in *miR-146a* expression downregulated *Smad4* and upregulated *VEGF* expression and induced the apoptosis of the mechanically injured chondrocytes [53]. The anti-apoptotic effect of *C. glomerata* has already been demonstrated on ASCs_EMS_ as well as on ASCs exposed to H_2_O_2_; its methanolic extract thus remarkably reduced the transcription of *p53*, *Bax*, and *p21* factors, while stimulating the expression of the *Bcl-2* cell survival gene [19, 27]. In the present studies, similar results were observed after chondrogenesis of the same cell type, where extract strongly stimulated the survival of chondrogenic cells. In addition, the suppression of the expression of the two pro-apoptotic and hypertrophic factors *VEGF* and *miR-146a-5p* has reinforced the beneficial effect of the extract. Mitochondrial dysfunction has been widely implicated in the mediation of several pathways implicated in cartilage degradation [54]. Chondrogenic EMS cells exhibited marked collapse of mitochondrial membrane potential as well as reduced number of active mitochondria evidenced by MitoRed staining as compared to healthy cells. Chondrocyte mitochondrial dysfunction is usually manifested by a decline in membrane potential and activity of complexes I, II, and III, leading to the alteration of several pathways involved in the breakdown of cartilage metabolism, including oxidative stress, defective chondrocyte biogenesis, increased cytokine-induced inflammation and matrix catabolism, and increased apoptosis of chondrocytes [55]. Influence of oxidative stress on cartilage tissue is directly related to the involvement of ROS, nitric oxide (NO), peroxynitrite (ONOO−), and superoxide radical anion (O_2_) in cartilage degradation. Under normal physiological conditions, the deleterious effect of ROS is counterbalanced by the intervention of enzymatic and non-enzymatic antioxidants acting by inhibiting the oxidizing enzymes or by detoxifying the cells of the free radicals. Our investigation showed that ASCs_EMS_ chondrogenic cells were highly impaired in terms of endogenous antioxidant defenses as evidenced by downregulation of *Sod1*, *Sod2*, and *CAT* genes. Moreover, flow cytometry-based analysis established that cells from the untreated control group were prone to consequent production of intracellular ROS and NO. Many previous experiments already pointed out the fact that during the onset of metabolic syndrome in horse, ASCs are likely to overproduce ROS as a consequence of mitochondrial dysfunction, increased inflammation, and dysregulated lipid metabolism [18, 20, 21, 23]. As a consequence, resulting oxidative stress can trigger telomere genomic instability, replicative senescence, collapse of proper antioxidant capacities, and dysfunction of chondrocytes that probably lead to the development and progression of cartilaginous defects such as OA [56]. Application of *C. glomerata* methanolic extract significantly improved the metabolic status of ASCS_EMS_-derived chondrogenic cells through mitochondrial potentiation, inhibition of ROS and NO production, and stimulation of antioxidant enzyme transcription. These results are in concordance with our previous findings that have proven the efficiency of the macroalga *Cladophora glomerata* to overcome oxidative stress and mitochondrial defects in ASC cells [19, 27].

## Conclusion

Our data show that the different concentration conditions of *Cladophora glomerata* methanolic extract exhibited significant effect on the general condition of EqASCs_EMS_ cells. The results demonstrated that extract exerts an obvious effect on stimulating the cell proliferation of ASCs in vitro. It also highlighted a marked potential for promoting the induced chondrogenic differentiation of impaired ASCs_EMS_ in vitro. Almost all markers of chondrogenesis involved in the formation of the cartilage matrix and in the regulation of the process have been at least restored or upregulated after stimulation with extract. In addition, a minimization of the hypertrophic process via the repression of the triggering factors, the reduction of apoptosis, and improvement of the mitochondrial condition has been observed. These effects of *Cladophora glomerata* may facilitate and improve the potential use of EqASCs for cartilage repair and regeneration.

## Data Availability

All datasets generated and/or analyzed during the current study are presented in the article, the accompanying Source Data or Supplementary Information files, or are available from the corresponding author upon reasonable request.

## Abbreviations

7-AAD: 7-Aminoactinomycine D
ACAN: Aggrecan
*ADAMTS-5*: *A disintegrin and metalloproteinase with thrombospondin motifs 5*
Agc1: Aspartate/Glutamate Carrier 1
ALP: Alkaline phosphatase
AP-1: Antigen Activator Protein 1
ASCs: Adipose-derived stromal stem cells
BAX: Bcl-2-associated X protein
BAX: BCL-2-associated X protein
Bcl2: B cell lymphoma 2
BCL-2: B cell lymphoma 2
BMP: Bone morphogenetic protein
BrdU: Bromodeoxyuridine
Cal2A1: Collagen type II alpha 1 chain
Casp9: Caspase 9
CD44: Homing cell adhesion molecule
*CD-rap*: *Cartilage-derived retinoic acid-sensitive protein*
CFU-fs: Colony-forming unit-fibroblasts
*Col11a2*: *Collagen type XI alpha 2 chain*
*Col9a1*: *Collagen type IX alpha 1 chain*
COMP: Cartilage oligomeric matrix protein
DAPI: 4′,6-Diamidyno-2-fenyloindol
DMEM: Dulbecco’s modified Eagle’s medium
ECM: Extracellular matrix
EMS: Equine metabolic syndrome
ER: Endoplasmic reticulum
FBS: Fetal bovine serum
GAPDH: Glyceraldehyde 3-phosphate dehydrogenase
HA: Hyaluronic acid
HBSS: Hank’s Balanced Salt Solution
HIF-2α: Hypoxia-Inducible Factors 2 Alpha
HRP: Horseradish peroxidase
IHH: Indian Hedgehog Homolog
JNK: c-Jun N-terminal kinase
MMP: Mitochondrial membrane potential
MMPs: Matrix metalloproteinases
MSCs: Mesenchymal stem cells
Nanog: Nanog homeobox
NF-κB: Nuclear Factor kappa-light-chain-enhancer of activated B cells
NO: Nitric oxide
OA: Osteoarthritis
Oct-4: Octamer-binding transcription factor 4
p21: Cyclin dependent kinase inhibitor 1A
p53: Tumor suppressor p53
PFA: Paraformaldehyde
PS: Penicillin–streptomycin
PTHrP-HDAC4: Parathyroid Hormone-Related Protein - Histone Deacetylase 4
RNS: Reactive nitrogen species
ROS: Reactive oxygen species
RT-qPCR: Quantitative reverse transcription polymerase chain reaction
Runx-2: Runt-related transcription factor 2
SEM: Scanning electron microscopy
Sod1 (Cu/Zn SOD): Copper-zinc-dependant superoxide dismutase (CuZnSOD)
Sod2 (Mn SOD): Manganese-dependent superoxide dismutase (MnSOD)
Sox2: Sex determining region Y-box 2
Sox5: Sex determining region Y-box 5
Sox6: Sex determining region Y-box 6
Sox-9: Sex determining region Y-box 9
TGF-β: Transforming growth factor beta
TNF-α: Tumor necrosis factor alpha
VEGF: Vascular endothelial growth factor
Wnt: Drosophila gene wingless and int derived from the proto-oncogene integration-1

## References

[1] Cokelaere S, Malda J, van Weeren R (2016). Cartilage defect repair in horses: current strategies and recent developments in regenerative medicine of the equine joint with emphasis on the surgical approach. Vet J.

[2] Desjardins MR, Hurtig MB (1990). Cartilage healing: A review with emphasis on the equine model. Can Vet J.

[3] Han Y, Lefebvre V (2008). L-Sox5 and Sox6 drive expression of the aggrecan gene in cartilage by securing binding of Sox9 to a far-upstream enhancer. Mol Cell Biol.

[4] Meina Wang, Jie Shen, Hongting Jin, Hee-Jeong Im, John Sandy, Di Chen. (2013). Cartilage Degeneration During Osteoarthritis, 61–69. doi:10.1111/j.1749-6632.2011.06258.x.Recent 10.1111/j.1749-6632.2011.06258.x PMC3671949 22172041

[5] Arbor A, Program BC (2007). Differential signal transduction of alternatively spliced FGFR2 variants expressed in human mammary epithelial cells. J Cell Physiol.

[6] Stanton H, Melrose J, Little CB, Fosang AJ (2011). Proteoglycan degradation by the ADAMTS family of proteinases. Biochim Biophys Acta Mol basis Dis.

[7] Zhuo Q, Yang W, Chen J, Wang Y (2012). Metabolic syndrome meets osteoarthritis. Nat Rev Rheumatol.

[8] Gregor MF, Hotamisligil GS (2011). Inflammatory mechanisms in obesity. Annu Rev Immunol.

[9] Monteiro Rosário, Azevedo Isabel (2010). Chronic Inflammation in Obesity and the Metabolic Syndrome. Mediators of Inflammation.

[10] Chen D, Shen J, Zhao W, Wang T, Han L, Hamilton JL, Im HJ. Osteoarthritis: toward a comprehensive understanding of pathological mechanism. Bone Research. 2017;5(August 2016). 10.1038/boneres.2016.44. 10.1038/boneres.2016.44 PMC5240031 28149655

[11] Kalamegam G, Memic A, Budd E, Abbas M, Mobasheri A (2018). A comprehensive review of stem cells for cartilage regeneration in osteoarthritis. Adv Exp Med Biol.

[12] Pearle AD, Warren RF, Rodeo SA (2005). Basic science of articular cartilage and osteoarthritis. Clin Sports Med.

[13] Lee PT, Li WJ (2017). Chondrogenesis of embryonic stem cell-derived mesenchymal stem cells induced by TGFβ1 and BMP7 through increased TGFβ receptor expression and endogenous TGFβ1 production. J Cell Biochem.

[14] Merckx G, Bronckaers A, Hilkens P, Ratajczak J, Lo Monaco M, Clegg P (2018). Stem cells for cartilage repair: preclinical studies and insights in translational animal models and outcome measures. Stem Cells Int.

[15] Baldari Silvia, Di Rocco Giuliana, Piccoli Martina, Pozzobon Michela, Muraca Maurizio, Toietta Gabriele (2017). Challenges and Strategies for Improving the Regenerative Effects of Mesenchymal Stromal Cell-Based Therapies. International Journal of Molecular Sciences.

[16] Leri SD, A. (2008). Aging and disease as modifiers of efficacy of cell therapy. Circ Res.

[17] Marycz K, Kornicka K, Szlapka-Kosarzewska J, Weiss C (2018). Excessive endoplasmic reticulum stress correlates with impaired mitochondrial dynamics, mitophagy and apoptosis, in liver and adipose tissue, but not in muscles in EMS horses. Int J Mol Sci.

[18] Kornicka K, Houston J, Marycz K (2018). Dysfunction of mesenchymal stem cells isolated from metabolic syndrome and type 2 diabetic patients as result of oxidative stress and autophagy may limit their potential therapeutic use. Stem Cell Rev Rep.

[19] Marycz K, Michalak I, Kocherova I, Edziak MM, Weiss C (2017). The cladophora glomerata enriched by biosorption process in Cr(III) improves viability, and reduces oxidative stress and apoptosis in equine metabolic syndrome derived adipose mesenchymal stromal stem cells (ASCs) and their extracellular vesicles (MV’s). Marine Drugs.

[20] Marycz Krzysztof, Kornicka Katarzyna, Basinska Katarzyna, Czyrek Aleksandra (2016). Equine Metabolic Syndrome Affects Viability, Senescence, and Stress Factors of Equine Adipose-Derived Mesenchymal Stromal Stem Cells: New Insight into EqASCs Isolated from EMS Horses in the Context of Their Aging. Oxidative Medicine and Cellular Longevity.

[21] Nawrocka D, Kornicka K, Śmieszek A, Marycz K (2017). Spirulina platensis improves mitochondrial function impaired by elevated oxidative stress in adipose-derived mesenchymal stromal cells (ASCs) and intestinal epithelial cells (IECs), and enhances insulin sensitivity in equine metabolic syndrome (EMS) horse. Marine Drugs.

[22] Marycz K, Weiss C, Śmieszek A, Kornicka K (2018). Evaluation of oxidative stress and mitophagy during adipogenic differentiation of adipose-derived stem cells isolated from equine metabolic syndrome (EMS) horses. Stem Cells Int.

[23] Marycz K, Kornicka K, Marędziak M, Golonka P, Nicpoń J (2016). Equine metabolic syndrome impairs adipose stem cells osteogenic differentiation by predominance of autophagy over selective mitophagy. J Cell Mol Med.

[24] Udalamaththa VL, Jayasinghe CD, Udagama PV (2016). Potential role of herbal remedies in stem cell therapy: proliferation and differentiation of human mesenchymal stromal cells. Stem Cell Research Therapy.

[25] Sabeena Farvin KH, Jacobsen C (2013). Phenolic compounds and antioxidant activities of selected species of seaweeds from Danish coast. Food Chem.

[26] Srimaroeng C, Ontawong A, Saowakon N, Vivithanaporn P, Pongchaidecha A, Amornlerdpison D (2015). Antidiabetic and renoprotective effects of Cladophora glomerata Kützing extract in experimental type 2 diabetic rats: a potential nutraceutical product for diabetic nephropathy. J Diabetes Res.

[27] Bourebaba L, Michalak I, Röcken M, Marycz K (2019). Cladophora glomerata methanolic extract decreases oxidative stress and improves viability and mitochondrial potential in equine adipose derived mesenchymal stem cells (ASCs). Biomed Pharmacother.

[28] Hudson JB, Kim JH, Lee MK, DeWreede RE, Hong YK (1998). Antiviral compounds in extracts of Korean seaweeds: evidence for multiple activities. J Appl Phycol.

[29] Suszynska M, Poniewierska-Baran A, Gunjal P, Ratajczak J, Marycz K, Kakar SS (2014). Expression of the erythropoietin receptor by germline-derived cells - further support for a potential developmental link between the germline and hematopoiesis. J Ovarian Res.

[30] Wang CY, Chen LL, Kuo PY, Chang JL, Wang YJ, Hung SC (2010). Apoptosis in chondrogenesis of human mesenchymal stem cells: effect of serum and medium supplements. Apoptosis.

[31] Cooper KL, Oh S, Sung Y, Dasari RR, Kirschner MW, Tabin CJ (2013). Multiple phases of chondrocyte enlargement underlie differences in skeletal proportions. Nature.

[32] Akiyama H, Chaboissier MC, Martin JF, Schedl A, de Crombrugghe B (2002). The transcription factor Sox9 has essential roles in successive steps of the chondrocyte differentiation pathway and is required for expression of Sox5 and Sox6. Genes Dev.

[33] Zwickl H, Niculescu-Morzsa E, Halbwirth F, Bauer C, Jeyakumar V, Reutterer A (2016). Correlation analysis of SOX9, -5, and -6 as well as COL2A1 and Aggrecan gene expression of collagen I implant–derived and osteoarthritic chondrocytes. Cartilage.

[34] Maldonado M, Nam J (2013). The role of changes in extracellular matrix of cartilage in the presence of inflammation on the pathology of osteoarthritis. Biomed Res Int.

[35] Wang L, Diao H, Zhou H, Li X, Chen Q, Jiang Z (2013). Cartilage oligomeric matrix protein (COMP)-mediated cell differentiation to proteolysis mechanism networks from human normal adjacent tissues to lung adenocarcinoma. Anal Cell Pathol.

[36] Strong AL, Gimble JM, Bunnell BA (2015). Analysis of the pro- and anti-inflammatory cytokines secreted by adult stem cells during differentiation. Stem Cells Int.

[37] Oh Chun-do, Lu Yue, Liang Shoudan, Mori-Akiyama Yuko, Chen Di, de Crombrugghe Benoit, Yasuda Hideyo (2014). SOX9 Regulates Multiple Genes in Chondrocytes, Including Genes Encoding ECM Proteins, ECM Modification Enzymes, Receptors, and Transporters. PLoS ONE.

[38] Gris, D. (2013). Public Access NIH Public Access, 185(2), 974–981. doi:10.1038/mp.2011.182.doi.

[39] Papaioannou G, Mirzamohammadi F, Lisse TS, Nishimori S, Wein MN, Kobayashi T (2015). MicroRNA-140 provides robustness to the regulation of hypertrophic chondrocyte differentiation by the PTHrP-HDAC4 pathway. J Bone Miner Res.

[40] Xu J, Lv S, Hou Y, Xu K, Sun D, Zheng Y (2018). miR-27b promotes type II collagen expression by targetting peroxisome proliferator-activated receptor-γ2 during rat articular chondrocyte differentiation. Bioscience Reports.

[41] Zhong L, Huang X, Karperien M, Post JN (2015). The regulatory role of signaling crosstalk in hypertrophy of MSCs and human articular chondrocytes. Int J Mol Sci.

[42] Selvamurugan N, Jefcoat SC, Kwok S, Kowalewski R, Tamasi JA, Partridge NC (2006). Overexpression of Runx2 directed by the matrix metalloproteinase-13 promoter containing the AP-1 and Runx/RD/Cbfa sites alters bone remodeling in vivo. J Cell Biochem.

[43] Lu H, Lin Z, Yang Z, Chen M, Zhang K (2016). Inhibition of RUNX2 expression promotes differentiation of MSCs correlated with SDF-1 up-regulation in rats. Int J Clin Exp Pathol.

[44] Yang B, Guo H, Zhang Y, Chen L, Ying D, Dong S (2011). Microrna-145 regulates chondrogenic differentiation of mesenchymal stem cells by targeting SOX9. PLoS One.

[45] Paik S, Jung HS, Lee S, Yoon DS, Park MS, Lee JW (2012). miR-449a regulates the chondrogenesis of human mesenchymal stem cells through direct targeting of lymphoid enhancer-binding Factor-1. Stem Cells Dev.

[46] Ahmed Salahuddin (2010). Green tea polyphenol epigallocatechin 3-gallate in arthritis: progress and promise. Arthritis Research & Therapy.

[47] Khanna D, Sethi G, Ahn KS, Pandey MK, Kunnumakkara AB, Sung B (2007). Natural products as a gold mine for arthritis treatment. Curr Opin Pharmacol.

[48] Thirunavukkarasu K, Pei Y, Moore TL, Wang H, Yu XP, Geiser AG, Chandrasekhar S (2006). Regulation of the human ADAMTS-4 promoter by transcription factors and cytokines. Biochem Biophys Res Commun.

[49] Khalifé S, Zafarullah M (2011). Molecular targets of natural health products in arthritis. Arthritis Res Ther.

[50] Blanco FJ, Guitian R, Vázquez-Martul E, De Toro FJ, Galdo F (1998). Osteoarthritis chondrocytes die by apoptosis: A possible pathway for osteoarthritis pathology. Arthritis Rheum.

[51] Gerber HP, Vu TH, Ryan AM, Kowalski J, Werb Z, Ferrara N (1999). VEGF couples hypertrophic cartilage remodeling, ossification and angiogenesis during endochondral bone formation. Nat Med.

[52] Miyaki S, Asahara H (2013). Macroview of microRNA’s function on osteoarthritis. Nat Rev Rheumatol.

[53] Jin L, Zhao J, Jing W, Yan S, Wang X, Xiao C, Ma B (2014). Role of miR-146a in human chondrocyte apoptosis in response to mechanical pressure injury in vitro. Int J Mol Med.

[54] Ruiz-Romero C, Calamia V, Mateos J, Carreira V, Martínez-Gomariz M, Fernández M, Blanco FJ (2009). Mitochondrial dysregulation of osteoarthritic human articular chondrocytes analyzed by proteomics. Mol Cell Proteomics.

[55] Blanco FJ, López-Armada MJ, Maneiro E (2004). Mitochondrial dysfunction in osteoarthritis. Mitochondrion.

[56] Regan E, Flannelly J, Bowler R, Tran K, Nicks M, Carbone BD (2005). Extracellular superoxide dismutase and oxidant damage in osteoarthritis. Arthritis Rheum.

